# Magnetic, Electrical, and Physical Properties Evolution in Fe_3_O_4_ Nanofiller Reinforced Aluminium Matrix Composite Produced by Powder Metallurgy Method

**DOI:** 10.3390/ma15124153

**Published:** 2022-06-11

**Authors:** Negin Ashrafi, Azmah Hanim Mohamed Ariff, Dong-Won Jung, Masoud Sarraf, Javad Foroughi, Shamsuddin Sulaiman, Tang Sai Hong

**Affiliations:** 1 Department of Mechanical and Manufacturing Engineering, Faculty of Engineering, Universiti Putra Malaysia (UPM), Serdang 43400, Malaysia; ashrafinegin2000@gmail.com (N.A.); shamsuddin@upm.edu.my (S.S.); saihong@upm.edu.my (T.S.H.); 2 Research Center Advance Engineering Materials and Composites (AEMC), Faculty of Engineering, Universiti Putra Malaysia (UPM), Serdang 43400, Malaysia; 3 Department of Mechanical Engineering, Jeju National University, 1 Ara 1-dong, Jeju 690-756, Korea; 4 Deputy Vice Chancellor’s Office (Research & Innovation), University of Malaya, Kuala Lumpur 50603, Malaysia; masoudsarraf@gmail.com; 5 Materials Science and Engineering Department, Sharif University of Technology, Azadi Avenue, Tehran P.O. Box 11155-9466, Iran; 6 School of Mechanical & Manufacturing Engineering, The University of New South Wales, Sydney, NSW 2052, Australia; foroughi@uow.edu.au

**Keywords:** magnetic iron oxide nanoparticles, thermal properties, magnetic properties, electrical properties, aluminium matrix composite, powder metallurgy

## Abstract

An investigation into the addition of different weight percentages of Fe_3_O_4_ nanoparticles to find the optimum wt.% and its effect on the microstructure, thermal, magnetic, and electrical properties of aluminum matrix composite was conducted using the powder metallurgy method. The purpose of this research was to develop magnetic properties in aluminum. Based on the obtained results, the value of density, hardness, and saturation magnetization (Ms) from 2.33 g/cm^3^, 43 HV and 2.49 emu/g for Al-10 Fe_3_O_4_ reached a maximum value of 3.29 g/cm^3^, 47 HV and 13.06 emu/g for the Al-35 Fe_3_O_4_ which showed an improvement of 41.2%, 9.3%, and 424.5%, respectively. The maximum and minimum coercivity (Hc) was 231.87 G for Al-10 Fe_3_O_4_ and 142.34 G for Al-35 Fe_3_O_4_. Moreover, the thermal conductivity and electrical resistivity at a high weight percentage (35wt.%) were 159 w/mK, 9.9 × 10^−4^ Ω·m, and the highest compressive strength was 133 Mpa.

## 1. Introduction

Aluminium matrix composite (AMC) has a distinctive characteristic in multifunctional electronic packaging, renewable energy, optoelectronic devices, the telecommunication industry, medical equipment, access control systems, and others [1]. However, many researchers have confirmed that AMC is an applicable composite to improve magnetic properties in various applications, such as electrical, aeronautical, and automotive applications [2,3]. Aluminium is categorised as a paramagnetic alloy with poor magnetic properties compared to ferrous materials such as steel, titanium, cast iron, and carbon steel [4]. The innovation of new magnetic composite materials with continuous development has resulted in various choices of superconducting permanent magnet applications in electromagnetic shielding or absorption [5].

Magnetite (Fe_3_O_4_), on the other hand, is a natural iron oxide magnet, which is the most abundant magnetic mineral on earth. It is one of the important ores of iron. A significant property of magnetite is the Verwey transition (Tv » 120 K), which results from a small distortion in the crystal structure when it changes from inverse cubic spinel to monoclinic and the effect presents itself in the electrical conductivity reduction, thermal expansion, and magnetisation of the mineral at the transition point [6,7]. Magnetite is believed to be an appropriate filler because it is abundant, inexpensive, and produces high free energy upon reaction with aluminium. This reaction develops wettability among aluminium and magnetite in providing additional energy for the rest of the process [8]. Magnetite nanoparticles are favoured for their paramount characterised filler materials because of their noble magnetic properties. In addition, ferromagnetic materials can be classified as “soft” or “hard” depending on their response to an external magnetic field. Hard magnetic materials have a wide hysteresis loop, high saturation induction (Bs), large coercivity (Hc), and high residual induction (Br). On the other hand, soft magnetic materials have a narrow hysteresis loop, low Hc, and high magnetic permeability. Soft magnetic materials with a narrow hysteresis loop and low losses can be used as cores in electronic devices, such as power transformers in AC adapters [9,10].

Investigations on aluminium composite characteristics focusing on magnetic properties revealed that Fe_3_O_4_ nanoparticles led to an improvement in soft magnetic properties [11,12,13].

Ferreira et al. investigated the magnetic properties of AMC reinforced with 10, 20, and 30 wt. % Fe_3_O_4_ using the powder metallurgy method. It was observed that the Ms of the composites increased by increasing the weight percentage of Fe_3_O_4_. Meanwhile, the saturation magnetisation (Ms) of Al-10F (0.55 ± 0.02 emu/g) and Al-20F (7.46 ± 0.05 emu/g) (Ferreira et al., 2016) was achieved with the addition of silicon carbide in the experiment, and it was observed that the Ms improved compared to the Ms for the Al-10 Fe_3_O_4_ of 0.55 emu/g [10].

Fathy et al. studied the impact of iron on the mechanical, microstructure, and magnetic properties of AMCs. Mechanical ball milling was utilised for the synthesis of 0.5%, 10%, and 15% Fe-Al composites. The outcomes demonstrated that the Hc for Al-15%Fe was higher than that of Al−5%Fe and Al−10%Fe composites. This might be ascribed to the moderately fine microstructure of the 15% Fe-Al composite [14].

Maleki et al. studied the addition of magnetic nickel ferrite into aluminium. A nickel ferrite-reinforced aluminium matrix should generate a lighter material with exceptional magnetic attributes for multiple applications, including sensitive measurement tools, automotive, and aeronautical purposes. As such, this study synthesised AMCs reinforced with NiFe_2_O_4_ nanoparticles to determine their magnetic characteristics, microstructure, and mechanical properties. Coercivity and magnetisation of the nanocomposite reinforced with 10 wt. % magnetic nanoparticles were 121 Oe and 1.7 emu/g, respectively [15].

Borgohain et al. fabricated magnetic composites with cobalt ferrite magnetic nanoparticles scattered in aluminium. Cobalt ferrite contributed to the significant development of the magnetisation estimation of the aluminium matrix with an Ms of 17.07 emu/g for the aluminium sample reinforced with 10 wt.% cobalt ferrite. The Ms and Hc of the composite with 1 wt.% cobalt ferrite were found to be 3.51 emu/g and 967 Oe, respectively, which changed to the Ms of 17.07 emu/g and Hc of 583 Oe with an increase in the ferrite content (10 wt.% cobalt ferrite) in the Al matrix [16].

Moreover, research on magnetic materials is mostly concentrated on achieving higher magnetic energy in a tiny size for various applications, such as transportation parts, hybrid motors, and sustainable energy technologies [17]. The thermal stability of modern permanent magnets is highly related to their resistance at elevated temperatures. It implies that permanent magnet variable flux sources are more efficient compared to electromagnets because of their small size; hence, large power supplies or cooling systems are not required [18]. It is also necessary to design advanced magnetic materials in electrical applications, such as automobiles and aircraft, to withstand higher speeds or high torsion, large loads, and higher temperatures of up to 600 °C. The invention of novel magnetic materials is an ongoing process, leading to a diverse selection of superconducting permanent magnet applications for the electrical industry [19,20].

There are limitations to design materials that can eliminate heat and preserve their dimensional stability at elevated temperatures and in corrosive environments in the electronics industry [21]. Although much research on the thermal properties of AMC has been conducted, there is no information available that focuses on the thermal properties of Al-Fe_3_O_4_ composites produced using the powder metallurgy method. Moreover, the thermal expansion coefficient of aluminium is higher compared to ceramics and metal oxides; thus, major variations in dimensions with increasing temperatures would cause difficulties with metallic components with close tolerance [22]. In this regard, one of the new methods to diminish the thermal expansion of aluminium is the addition of particles with a lower coefficient of thermal expansion, as reinforcing aluminium with Fe_3_O_4_ nanoparticles will lead to a decrease in thermal expansion.

AMCs are commonly manufactured, either using powder metallurgy or liquid route. However, the powder metallurgy method has been recognised as a very promising method and the most attractive option due to several advantages. One of the advantages is the control over the microstructural phases. The low temperature required throughout the process accounts for the severe control of interphase kinetics. Other fabrication techniques such as casting have the drawbacks of reinforcement segregation, reaction between reinforcement particles and matrix, interfacial chemical reactions, and high localised residual porosity along with poor interfacial bonding. Powder metallurgy can fabricate a homogeneous and net-shape product to obtain properties that are not achievable by conventional metal processing technologies [23].

Powder metallurgy is one of the low-cost methods for fabricating complex shapes. When magnetic powder is involved, interfacial reactions between magnetic powder and the aluminium matrix must be avoided. This is to inhibit the formation of undesirable phases that may reduce the bonding between the matrix and the particles, hence affecting its magnetisation property. On the other hand, porosity negatively affects mechanical and physical properties. The advantages of powder metallurgy include the ability to fabricate complex shapes with precise dimensions, uniform distribution of reinforcements, and lower porosity at low costs. The key advantage of powder metallurgy is that the reinforcement and matrix are mixed in the solid state which prevents the formation of any unwanted phases, the uniform distribution of reinforcement in the matrix and better control of microstructure, low manufacturing temperatures that reduces the interfacial reactions among the matrix and reinforcements.

There are various advantages of isostatic pressing over uniaxial pressing, including uniform strength in all directions. The pressure used to compact the powder is applied equally in all directions and provides uniform density, increases density, and improves the material’s mechanical properties. A two-step compaction results in lower porosity in samples. During the production of samples with the powder metallurgy technique, two factors need to be investigated, firstly the mechanical parameters such as compressing pressure, ball milling time and speed, sintering temperature, and time. Secondly, metallurgical factors such as the amount and shape (fiber or particles) of the reinforcement. Metallurgical factors refer to the metallurgical parameters such as the size and shape of the reinforcements, the interfacial characteristics, and also the volume fraction or weight percentage of the reinforcement [21,22,24,25,26].

In this study, aluminium composites that utilise Fe_3_O_4_ nanopowder were developed. The magnetic, electrical, thermal, hardness, and compressive properties are systematically investigated to identify the optimum amount of nanofiller with processing operational steps.

## 2. Material Selection and Methodology

### 2.1. Fabrication Procedure

Fe_3_O_4_ (40 nm), and Aluminium powder with a purity of 99.7% and an average grain size of 2 μm, purchased from (MHC Industrial Co., Ltd., Xiamen, China), was used to fabricate Al-Fe_3_O_4_ samples using the powder metallurgy method. Planetary ball mills (PM 100, Retsch, Haan, Germany) at room temperature were used for mixing the powder and binder. The powder and metal were mixed in a container at a continuous speed of 400 rpm for 1 h and in the reverse mixing direction for another hour. The weight ratio of milling ball-to-powder was 15:1. The powder mix with a binder (magnesium stearate) was placed into a tubular die with a diameter of 20 mm. The addition of a binder during ball milling can prevent the agglomeration of particles and refine the distribution of the reinforcement in the matrix. Laboratory conditions and sintering processes can produce a uniform mixture where microporosities appear between the reinforcement particles and the matrix. Once mixing was completed, a universal testing machine (Instron 3382, Shandong, China) was operated at a pressure of 250 MPa and a cold isostatic pressure (CIP) (Reiken Seiki, Niigata, Japan) of 250 kgf was applied for 15 min to compact the blended powder. The samples were heated at 600 °C for 20 min with a heating and cooling rate of 10 °C/min by a Linn High Therm furnace. Sintering was performed under an argon atmosphere to avoid oxidation throughout the sintering process. Five basic compositions were prepared in this study that rely on magnetic nano iron oxide. All of the compositions contained 5% Mg stearate powder. Five basic compositions of magnetic nano iron oxide were prepared (Table 1). The specimens were ground and polished using different abrasive papers of 800, 1200, 2000, and 2500 grit and diamond paste with alumina slurry, followed by ultrasonication in acetone and deionised water for 10 min to clean, and lastly drying was performed at 100 °C for 1 h. Keller’s reagent solution was used for 5 s to etch the surface of each sample prior to using an optical microscope (OM) and field emission scanning electron microscopy (FESEM, SU8000, Hitachi, Tokyo, Japan).

### 2.2. Phase and Microstructural Analysis

The phase analysis was completed using a PANalytical Empyrean system (grazing incidence X-ray diffraction (GIXRD), Almelo, The Netherlands) with Cu-Kα radiation (λ = 1.54178 Å) over a 2θ range from 20° to 80° operating at 45 kV and 30 mA, with a scanning rate of 0.1° s^−1^ and a step size of 0.026°. The PANalytical X’Pert HighScore software was utilised to determine the X-ray diffraction (XRD) patterns; therein, all the reflections corresponded with the standards given by the Joint Committee on Powder Diffraction and Standards (JCPDS, card 02-1109 for Al and 01-075-1609 for Fe_3_O_4_). To examine the microstructure and the morphologies of particles, FESEM (SU8000, Hitachi, Tokyo, Japan) with an acceleration voltage of 2 kV was applied. Energy-dispersive spectroscopy (EDS) connected to the FESEM device was used to carry out the component analysis.

### 2.3. Density Measurement

The density measurement was conducted using Archimedes’ principle, where the samples were first weighed in the air and then in water according to ASTM B311-08. The measured density of the composites was determined using water displacement, and the theoretical density of the composites was estimated by the rule of mixtures equation (Equation (1)). The rule of mixtures is a technique for the approximate calculation of composite properties on the basis of a hypothesis that a composite material’s property is the volume-weighted mean of the phases (matrix and reinforcement phases) properties.

(1) $$\rho_{c}=(\rho_{m}\times V_{m})+(\rho_{f}\times V_{f})+(\rho_{f}\times V_{f})$$

where $\rho_{c}$, $\rho_{m}$, and $\rho_{f}$ refer to the densities of the composite, matrix, and reinforcement, respectively, while V_m_ and V_f_ describe the volume fraction of the matrix and dispersed reinforcement, respectively [27].

### 2.4. Microhardness Evaluation

The microhardness of the composites was assessed by a Vickers microhardness testing instrument (Mitutoyo-AVK C200-Akashi Corporation, Kanagawa, Japan) using a selected load (98.07 mN) and a holding time of 15 s. The tests were performed on five random parts and the average value was measured. The relationship between the Vickers microhardness and the load (F)/area of trace (A) was calculated using Equation (2), where Hv is the Vickers microhardness, F is the applied load, and d is the diagonal length of the indentation [28].

(2) $$H_{v}=1854.4\;(\frac{F}{d^{2}})$$

### 2.5. Compressive Test

**Figure 1:**
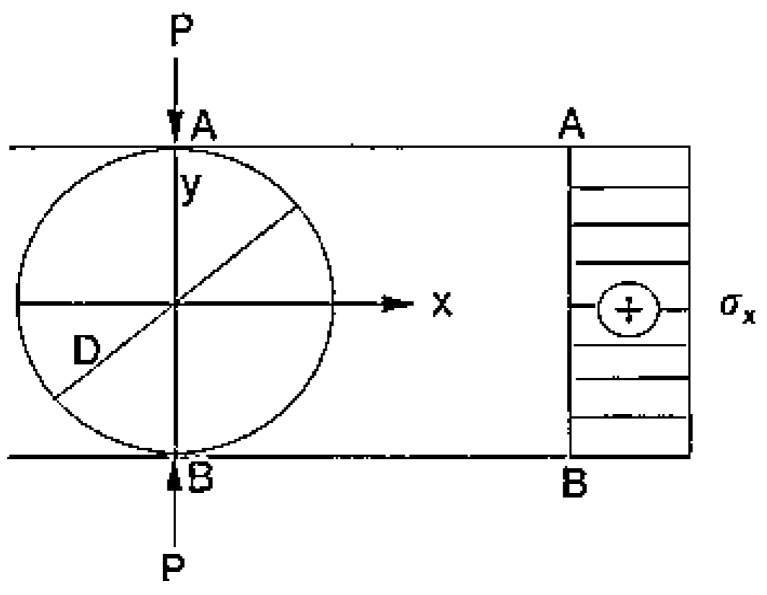
Schematic view of the diametral compression experiment.

The Brazilian test is a diametral compression experiment of cylindrical samples and is well-known for powder metallurgy, ceramic materials, and concrete. A diametral compression experiment includes implementing force until the material sample is broken in half. A schematic representation of the diametral compression experiment is presented in Figure 1 [29].

**Figure 2:**
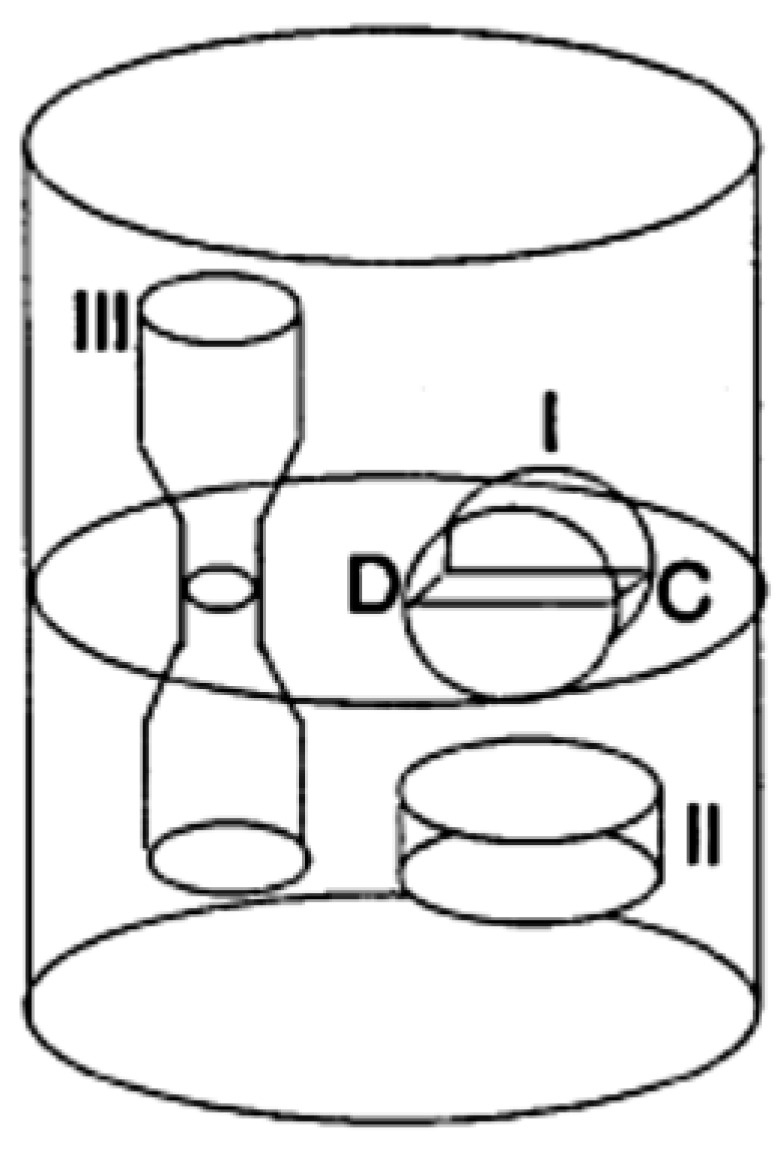
Tensile and diametral compression sample.

The samples were prepared as Type II in Figure 2. The diametral compression experiment was performed on discs of a 20 mm diameter and 5 mm thickness with a crosshead speed of 1 mm min^−1^ following ASTM E8/E8M.

The tensile strength can be calculated using Equation (3).

(3) $$\sigma_{x}=\frac{2P}{\pi \mathrm{Dt}}$$

where σ_x_ is the splitting tensile strength, P is the maximum applied load, t is the thickness of the specimen, and D is the diameter of the specimen [30].

### 2.6. Analysis of Magnetic Properties

A vibrating sample magnetometer device (VSM, Lakeshore 7407 series) was utilised to measure the magnetic response of the aluminium composite at room temperature. The measurements of magnetic properties, including the hysteresis loops and magnetisation with a vibrating sample magnetometer, were conducted. The coercive force, saturation magnetisation, and remanence of samples with a 2 mm width and 3 mm thickness were observed at an ambient temperature. For this purpose, the samples were cut into the desired size using a precision saw (IsoMet5000, Buehler, Leinfelden-Echterdingen, Germany).

### 2.7. Measurement of Electrical Properties

The four-point probe technique (Keithley Characterisation and Measurement Set) was utilised to measure the electrical properties of the composites, including resistivity and conductivity. For this purpose, 20 A current and 20 V voltage with direct current (DC) was employed at an ambient temperature. To achieve this objective, the sample dimension should be 10 mm in diameter. The electrical conductivity δ was calculated using Equation (4), where ρ is the electrical resistivity.

(4) $$\delta =\frac{1}{\rho }$$

There is a classical (model analysis) electrical conductivity equation that can be used to calculate electrical resistivity, which is equal to electrical conductivity. This can be achieved by replacing electrical resistivity with electrical conductivity, as in the Maxwell model (Equation (5)), where $\rho_{\mathrm{er}.c}$, $\rho_{\mathrm{er}.m}$, and $\rho_{\mathrm{er}.p}$, are the electrical resistivity of the composite, matrix, and particles, respectively, and $v_{p}$ is the volume fraction of particles.

(5) $$\rho_{\mathrm{er}.c}=\rho_{\mathrm{er}.m}\left[\frac{1+2\frac{\rho_{\mathrm{er}.m}}{\rho_{\mathrm{er}.p}}-2v_{p}\left(\frac{\rho_{\mathrm{er}.m}}{\rho_{\mathrm{er}.p}}-1\right)}{1+2\frac{\rho_{\mathrm{er}.m}}{\rho_{\mathrm{er}.p}}+2v_{p}\left(\frac{\rho_{\mathrm{er}.m}}{\rho_{\mathrm{er}.p}}-1\right)}\right]$$

### 2.8. Measurement of Thermal Properties

The linear thermal expansion coefficient α is identified as the fractional modification in the length of the sample ∆l/l per temperature degree modification ΔT(K) by Equation (6) [25].

(6) $$\alpha =\frac{1\;\Delta \mathrm{ls}}{\mathrm{ls}\;\Delta \;T}$$

The coefficient of thermal expansion (CTE) of a composite can be calculated from the matrix and fibre. When the matrix and fibres or particles are placed in a series, the change in length, ΔLc of the composite is given by the sum of the variants in the length of the components by Equation (7):

ΔLc = ΔL_1_ + ΔL_2_ (7)

where ΔL1 and ΔL2 are the variations in the dimensions of the component’s matrix and reinforcement, respectively. The difference in the expansion coefficients causes thermal stresses, which can be adequate to distort the matrix plastically, and consequently influences mechanical behaviour. In general, thermal diffusivity and thermal conductivity of composites increase by increasing the temperature. For the purpose of assessing the thermal diffusivity δ of cylinder samples (10 mm diameter) at room temperature, the laser flash method (LFA, Netzsch MicroFlash, LFA457, Selb, Germany) was employed, and the specific heat, C (J/gK) of the samples was computed by differential scanning calorimetry (DSC, Mettle Toledo, TGA/DSC 1 HT, Columbus, OH, USA). The thermal conductivity (W/mK) of the specimens was calculated by multiplying the thermal diffusivity, density, and specific heat capacity, as shown in Equation (8).

(8) $$\lambda =C_{p}\alpha \rho$$

where ρ is the density, α is the coefficient of thermal diffusivity, $C_{p}$ is the heat capacity, and λ is the thermal conductivity [26].

## 3. Results and Discussion

### 3.1. Microstructural Analysis

**Figure 3:**
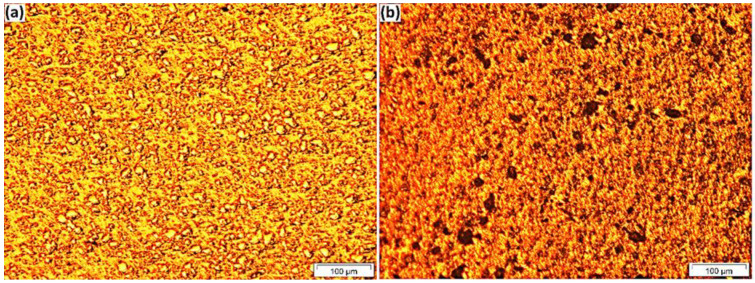
Optical microscopy for a.5 (**a**) before and (**b**) after 5-s etching.

The microstructure depends on several factors, such as solidification rate, type, and amount of reinforcement [27]. Figure 3 indicates the OM images of the a.5 composite before and after etching, which confirmed that the distribution of Fe_3_O_4_ particles in the composite is homogeneous, and no pores or cracks could be observed on the composite surface. The optical microscopy for a.5 identified that the microstructure consisted of the following three main phases: aluminium, Fe_3_O_4_, and intermetallic compound Al_3_Fe.

**Figure 4:**
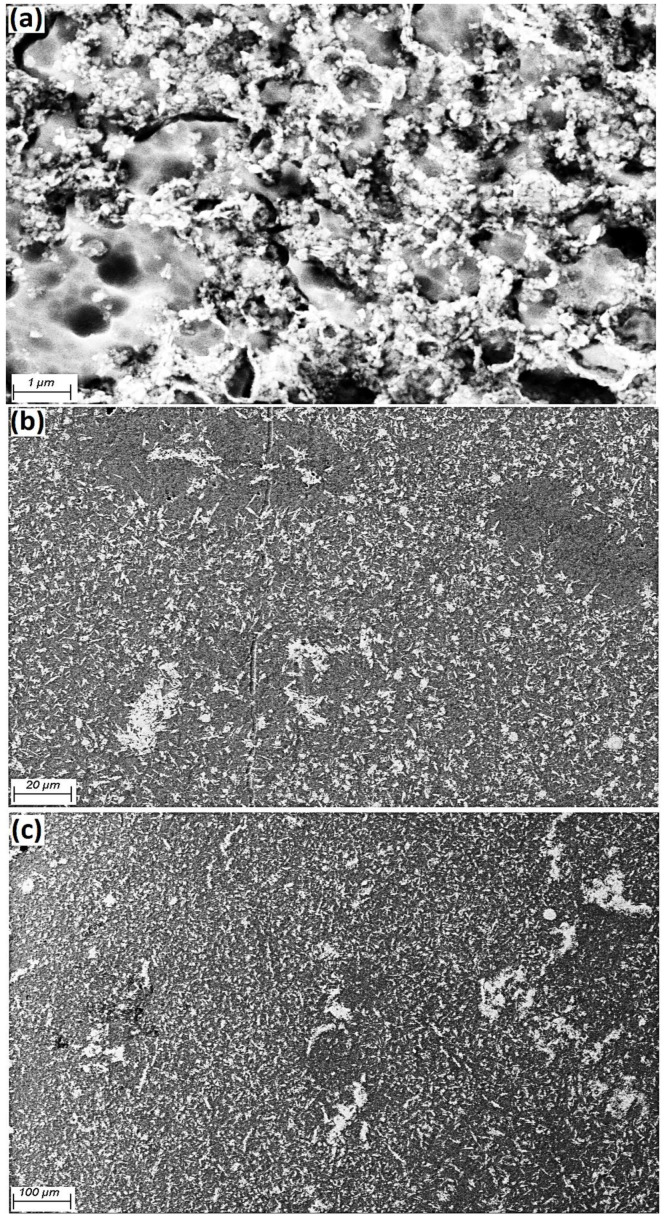
FESEM of a.5 composite, (**a**) 1 µm, (**b**) 20 µm, (**c**) 100 µm.

**Figure 5:**
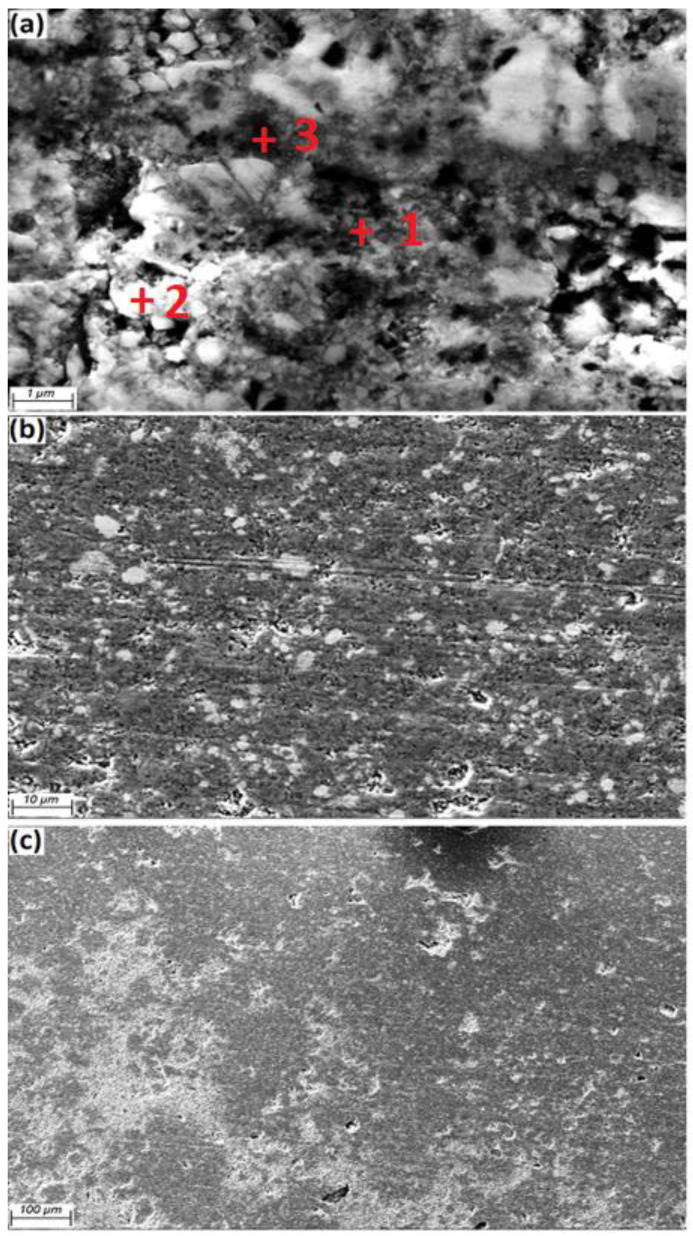
FESEM of a.5 composite sample after etching and heat treatment at 600 °C, (**a**) 1 µm, (**b**) 10 µm, (**c**) 100 µm.

Figure 4 shows the Al grains (grey colour) as a matrix and Fe_3_O_4_ particles (white colour, 40 nm) available at the Al grain boundaries. Additionally, the homogeneous scattering of particles in the matrix is evidently clarified. By increasing the amount of weight percentage of reinforcement, the likelihood of agglomeration at the grain boundaries also increased. FESEM micrography of the a.5 sample after etching and heat treatment at 600 °C indicated that the Al matrix and Fe_3_O_4_ powder (white) were placed at the grain boundaries, as presented in Figure 5.

Concerning soft magnetic properties, such as coercivity, permeability, and saturation magnetization; higher densities, lower quantities of interstitial sites, and larger grain sizes are required. Microstructural defects, such as dislocations and grain boundaries, tend to hinder the movement of domain walls, therefore enlarging and distorting the magnetization curve.

Both magnetic volume and grain size exert huge impacts on coercivity, density, DC losses, magnetic saturation, and mechanical strength, as observed for nanocrystalline and amorphous materials. Composites with larger grain and particle sizes can lower hysteresis losses and coercivities, and can enhance density due to certain defects (e.g., grain boundaries and airgaps) and limited nonmagnetic addition.

The larger grain size seemed to enhance magnetic performance, wherein both nanocrystalline and amorphous materials failed to outperform crystalline powder. As for the powder materials, if additional nonmagnetic regions were found between the particles, their impact on magnetic saturation and coercivity would be stronger than grain size. Particle boundaries may have more regions in terms of air gaps and thicker inclusions, which can lead to a larger demagnetizing field. Small particle sizes not only result in low density but also significantly more regions of boundaries due to low surface-to-volume ratios.

As mentioned before, the microstructure of materials has an impact on coercivity. Similarly, particle size and grain boundaries affect the hysteresis losses. SMCs that utilize organic binders are forced to use low-temperature curing, mainly for a breakdown in the coating consequences in strained ferrous grains and high hysteresis losses. Consequently, by using large-grained, higher-purity ferromagnetic powder, and post-compaction annealing to release impurities and work-hardened areas, the pinned domain walls can be removed and reduce the hysteresis losses.

**Figure 6:**
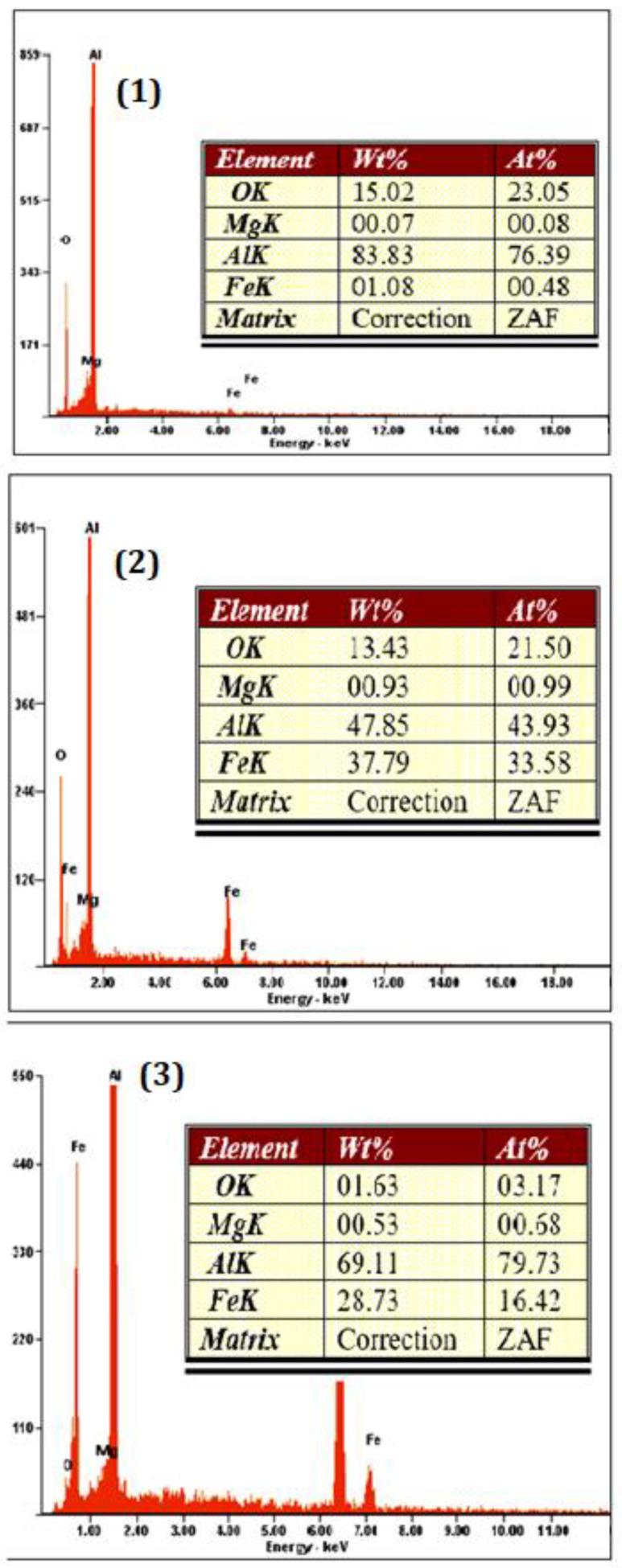
The EDS spectrum of a.5 composition (**1**) Al, (**2**) Fe_3_O_4,_ and (**3**) Al_3_Fe.

The EDS spectrum showed for a.5 after heat treatment at 600 °C. The EDS results in Figure 6 confirmed the presence of Al, Fe, O, and Mg in the grain boundaries. EDS analysis of the revealed surface displays the results for Point 1 is aluminum, where the Al peak (83.83 wt.%) is the main peak, and was detected by using FESEM, in a light gray color and Fe_3_O_4_ particles (white color) exists in the aluminium matrix, with confirmed peaks of Fe (47.85 wt.%) and O (37.79 wt.%) at point 2. The compositions of Al_3_Fe (Al 69.11 and Fe 28.73 wt.%) were detected at point 3, respectively, which were also confirmed by the XRD pattern.

### 3.2. X-ray Analysis

**Figure 7:**
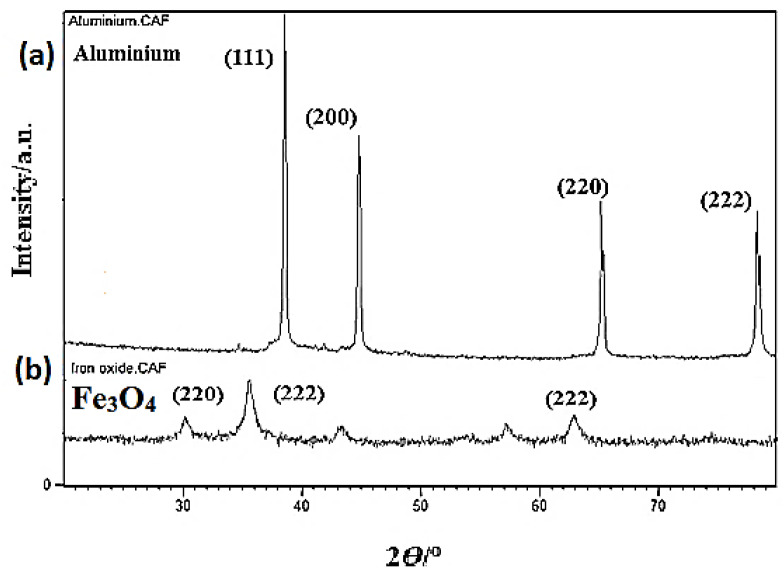
XRD analysis for (**a**) pure aluminium and (**b**) magnetite.

**Figure 8:**
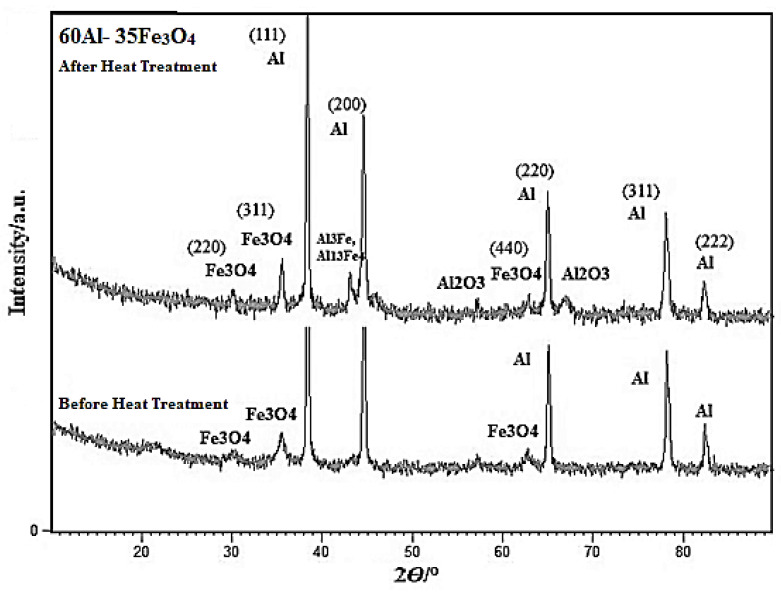
XRD analysis for the a.5 composite before and after sintering.

An X-ray analysis was performed before and after sintering. The XRD images of pure aluminium and Fe_3_O_4_ powder are displayed in Figure 7a,b. The XRD revealed four key peaks for aluminium (111, 200, 220, 222) as displayed in Figure 7a and three main peaks for Fe_3_O_4_ as illustrated in Figure 7b. Figure 8 indicates the XRD analysis of a.5 before and after heat treatment. The aluminium matrix has a face-centered cubic structure (FCC). After the addition of Fe_3_O_4_ nano-particles into aluminium, three peaks appeared, which refer to iron oxide with two crystal systems, Orthorhombic and Cubic, before heat treatment. Moreover, after heat treatment, three new peaks appeared which were assigned to Fe_3_O_4_ with the cubic phase. The XRD analysis of a.5 after heat treatment identified Al-Fe intermetallic compounds (Al_3_Fe, and Al_13_Fe_4_) and Al_2_O_3_. The first peak is related to Fe_3_O_4_ with a cubic crystallography system. After adding Fe_3_O_4_ nano-particles into aluminium, and heat treatment, five main peaks were revealed for aluminium, three main peaks for Fe_3_O_4_. 2θ = (38/784, 44/600, 65/186, 78/306, 82/352) were associated with aluminium, and 2θ = (30/125, 35/483, 62/629) was assigned to the Fe_3_O_4_ cubic crystal system, respectively.

### 3.3. Density Analysis

**Figure 9:**
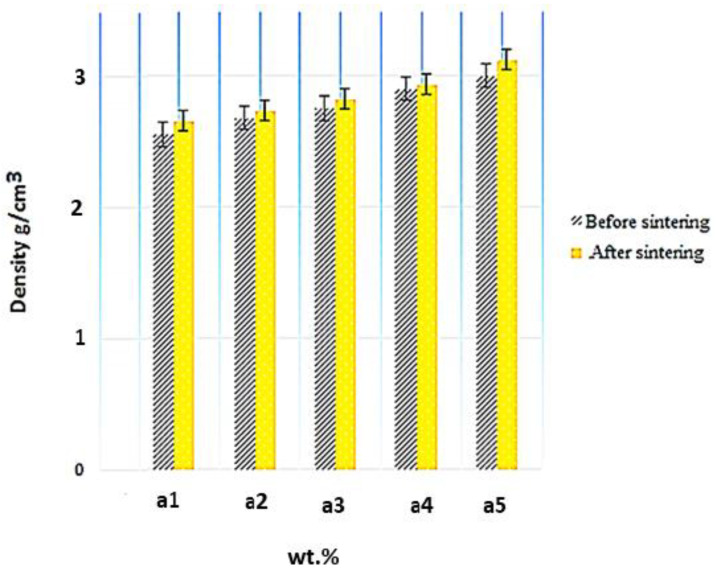
Evolution of density after and before sintering depending on the filler.

The density measurements were conducted using Archimedes’ principle [31]. As shown in Figure 9, the density differs from 2.33 to 3.29 g/cm^3^. The density values for Al-10 Fe_3_O_4,_ Al-10 Fe_3_O_4_, Al-15 Fe_3_O_4_, Al-20 Fe_3_O_4_, Al-30 Fe_3_O_4_, and Al-35 Fe_3_O_4_ are 2.33, 2.58, 2.74, 2.81, 2.92, and 2.98 g/cm^3^ before sintering and 2.67, 2.71, 2.79, 2.86, 2.97, and 3.29 g/cm^3^ after sintering, respectively, indicating a 41.2% improvement by increasing the weight percentage of Fe_3_O_4_. The density of magnetite (4.8 g/cm^3^) is higher than aluminium (2.70 g/cm^3^). The density measurements of the composites were performed using water displacement. The theoretical density of the composites was calculated with the rule of mixtures formula. By comparing the experimental and theoretical densities, the density of the composites were found to be acceptable. As can be seen, the density changed from 2.33 to 2.98 g/cm^3^ before sintering by increasing the weight percentage of Fe_3_O_4_ nanoparticles. Under the same condition, the density of Al-Fe_3_O_4_ composites after sintering was evaluated. After sintering, the density of each sample increased slightly. Figure 9 shows the evolution of density before and after sintering, where the density after sintering increased rapidly by 3.29 g/cm^3^ for Al-35 Fe_3_O_4_. Adding different amounts of reinforcements with a higher density increases the density of composites. Moreover, the density of the composites increases at high sintering temperatures. The diffusion of atoms is easier at higher sintering temperatures. As a result, the density of the composites increases.

**Figure 10:**
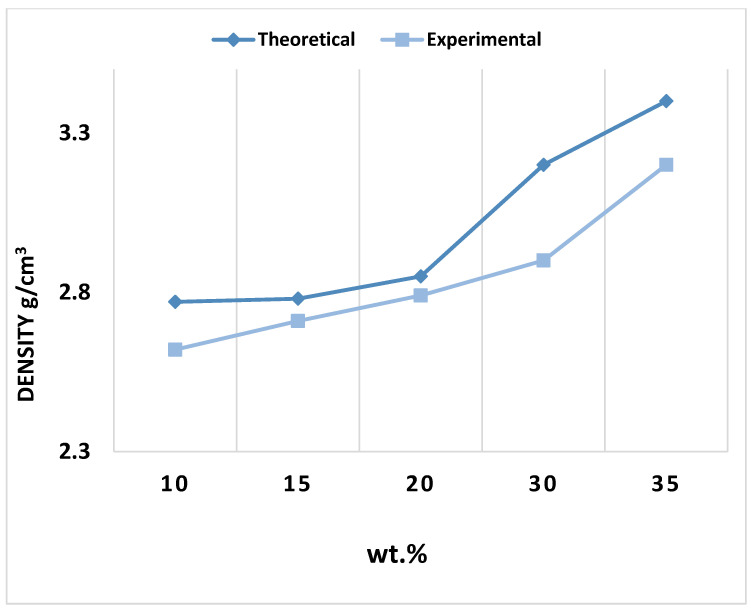
Theoretical and experimental densities of composites.

The density measurement for Al-Fe_3_O_4_ was performed using Archimedes’ principle. The samples were first weighed in the air and then in water according to ASTM B311-08. The density of the composites, as shown in Figure 9, varies slightly from 2.20 to 3.00 g/cm^3^. The density of the composites was calculated by water displacement, and the theoretical density of the composites was measured using the rule of mixtures equation. The density of the composites was calculated using Equation (9):

(9) $$\rho =\frac{m_{1}+}{\frac{m_{1}}{\rho_{1}}+}\frac{m_{2}}{\frac{m_{2}}{\rho_{2}}+}\frac{+m_{3}}{\frac{m_{3}}{\rho_{3}}}$$

where $m_{1}$ is the mass of the matrix and $m_{2}$ and $m_{3}$ are the mass of reinforcements. $\rho_{1}$ refers to the density of the matrix and $\rho_{2\;}\mathrm{and}\;\rho_{3}$ are the density of reinforcements. Figure 10 compares the theoretical and experimental densities of the composites. By comparing the theoretical density values, the density of the composites is found to be acceptable, with the relative density ranging from 90% to 98%. By dividing the density of the samples after sintering with the theoretical density, the relative density was determined to be between 90% and 98%.

### 3.4. Hardness Assessment

**Figure 11:**
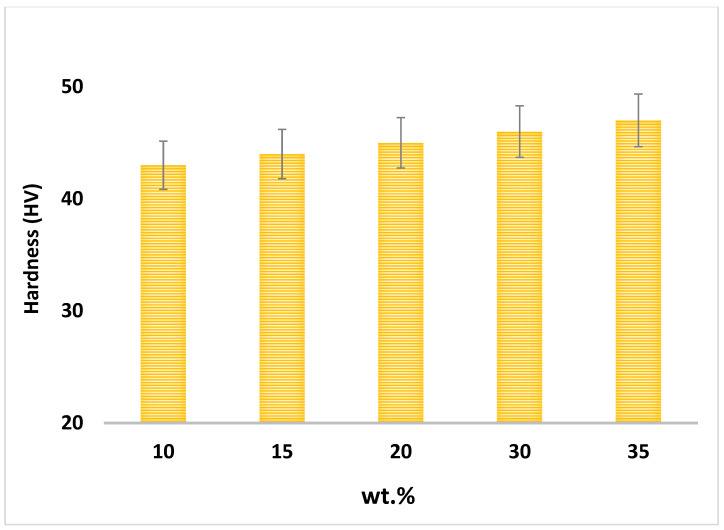
Vickers hardness values for different weight percentages of Fe_3_O_4_ in the composites.

The mechanical properties of composites depend on the weight percentage of reinforcement materials, sintering temperature, and microstructure [32]. Different weight percentages of nanofiller are added into the aluminium matrix to find an optimal amount of Fe_3_O_4_ powder and its properties, such as hardness, for composite modification. Therefore, the hardness of the composites with different weight percentages of Fe_3_O_4_ has been investigated. Figure 11 shows the samples’ hardness for different percentages of reinforcement particles in the matrix. The microhardness of the composites improved as the weight percentage of Fe_3_O_4_ nanoparticles increased to 35 wt.% Figure 11 shows that the highest hardness value (47 HV) belongs to the sample with 35 wt.% Fe_3_O_4_, while the hardness remained steady around 43 HV as a low amount of reinforcement (5–10 wt.%) was added. The hardness value improved by 9.3% from Al-5Fe_3_O_4_ to Al-35Fe_3_O_4_ composite. The iron oxide nanoparticles block the motion of dislocations and restrain the deformation of the nanocomposites, in addition to providing strong interfacial bonding between Al and ceramic nanoparticles, which are the key reasons for the improvement in microhardness [33].

### 3.5. Compressive Test

**Figure 12:**
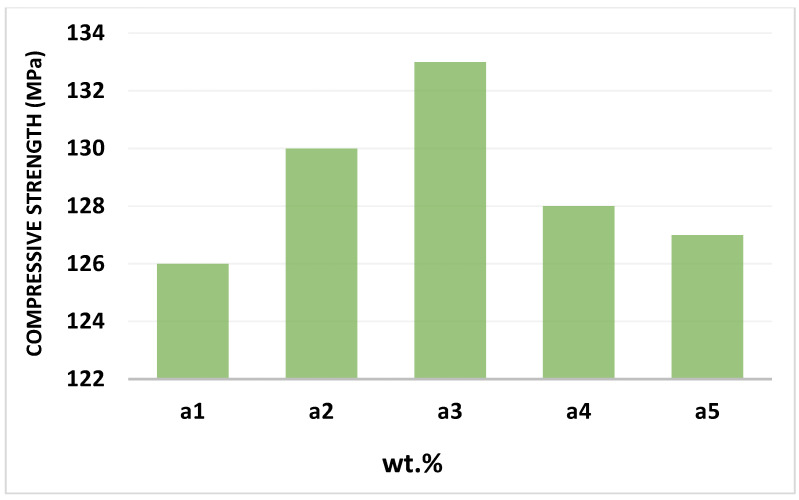
Compressive strength composite specimens with different weight percentages of reinforcement.

**Figure 13:**
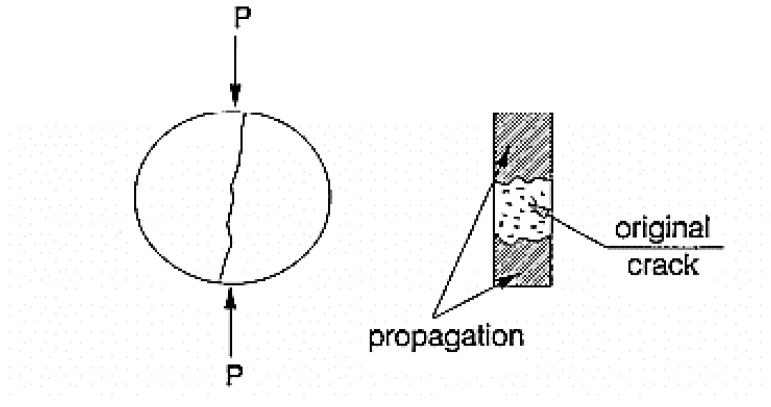
Scheme of the fracture in the Brazilian test.

The effect of the reinforcement on the compressive strength of Al-nano Fe_3_O_4_- composites fabricated by the cold isostatic pressure method is shown in Figure 12. As the weight percentage of Fe_3_O_4_ increased, the compressive strength associated with magnetite reinforcement initially improved and then remained constant. The reason for this increase is due to improved work-hardening, which can be due to the elastic properties of Fe_3_O_4_ particles, and as a result, prevents the plastic distortion of aluminium. The results confirmed the noticeable effect of Fe_3_O_4_ particles on the strengthening of the composites. In this regard, the strength increases as the amount of reinforcement increases. On the other hand, with an increasing amount of magnetite reinforcement particles, the homogeneous distribution of particles in the aluminium decreases, which may lead to cluster formation. Thus, Fe_3_O_4_ particles with low mechanical bonding between them reduce the compressive strength. A lack of bonding between the particles and the matrix alloy can reduce compressive strength. A decrease in compressive strength occurred slightly with an increase in the weight percentage of Fe_3_O_4_ particles in aluminium. The crack started in the central section of each disc sample, which is the most extremely stressed tension zone (Figure 13).

### 3.6. Magnetic Properties of Al-Fe_3_O_4_ Composites

Magnetised materials are named ferromagnetic (or ferrimagnetic). These materials contain nickel, iron, cobalt, and their alloys, with some alloys of rare-earth metals. Magnetism is classified as a physical phenomenon that is mediated by a magnetic field. The source of magnetism is the orbital and spin movement of electrons and how the electrons interact with each other. The magnetic behaviour of a material is related to different factors, such as pressure and temperature. Materials may reveal more than one form of magnetism. The magnetic action of materials can be categorised into diamagnetism, paramagnetism, ferromagnetism, and antiferromagnetism. Diamagnetism and paramagnetism materials exhibit no collective magnetic interactions, whereas ferromagnetism and antiferromagnetism materials indicate long-range magnetic order under a particular temperature [34]. Ferreira et al. synthesised Al-10 Fe_3_O_4_ with a particle size of 70 nm using a microwave and investigated its magnetic and Hc properties, which were 0.55 emu/g and 199 G, respectively. Therefore, Al-Fe_3_O_4_ composites are considered ferromagnetic [10].

**Figure 14:**
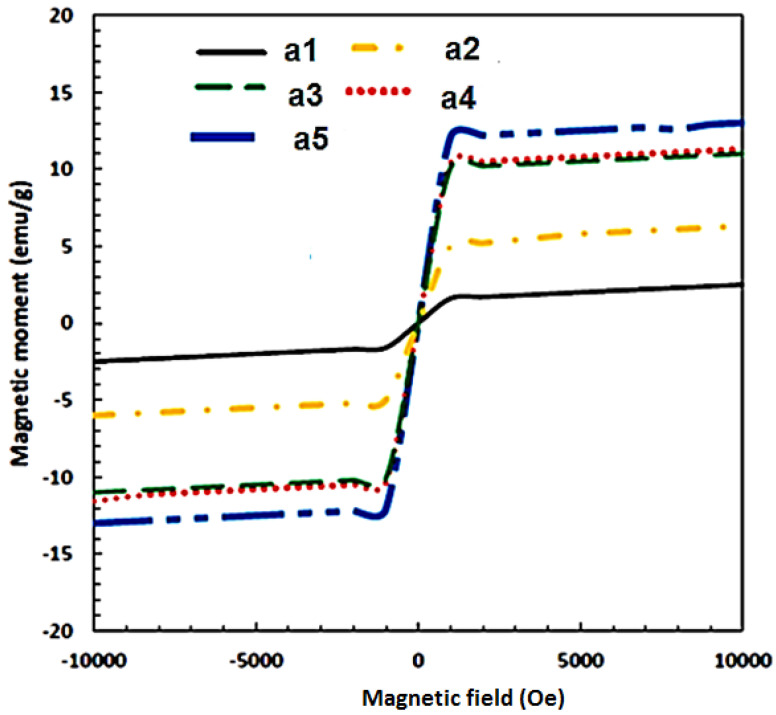
Magnetic hysteresis curve for Al-Fe_3_O_4_ with different weight percentages at ambient temperature.

**Figure 15:**
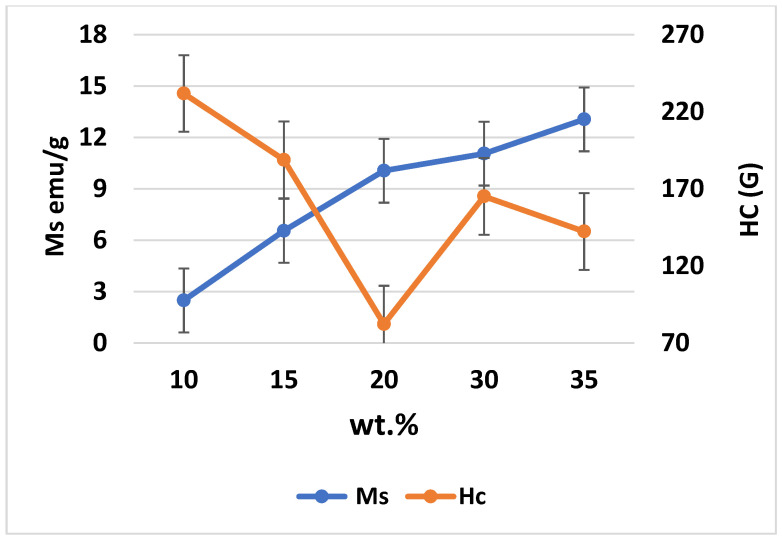
Correlation between Hc and Ms.

Figure 14 and Figure 15 present the magnetic hysteresis curve (N) based on the magnetic moment and field, as well as the saturation coercive field (Hc) and Ms for different weight percentages of Fe_3_O_4_ in the composites. The values of Ms and Hc varied with increasing weight percentages of Fe_3_O_4_, where the highest Ms was achieved at 35% Fe_3_O_4_. Based on the results, the Ms values are 2.49, 6.55, 10.06, 11.06, and 13.06 emu/g for Al-10 Fe_3_O_4_, Al-15 Fe_3_O_4_, Al-20 Fe_3_O_4_, Al-30 Fe_3_O_4_, and Al-35 Fe_3_O_4_, respectively. The Ms increased while the Hc decreased as the weight percentage of Fe_3_O_4_ increased. It means that Hc leads to an opposite trend as opposed to Ms, as shown in Figure 14. The curves indicated that the values of Hc did not fluctuate with a lower amount of iron oxide (10–15 wt.%). The maximum Hc is 231.87 G for Al-10 Fe_3_O_4_ and the Hc decreased to 188.82 G as the weight percentage of Fe_3_O_4_ increased from 10 to 15 wt.%. The addition of 15–20 wt.% Fe_3_O_4_ resulted in 150.26 G. Coercivity reduces due to the growth of the soft magnetic phase. It is proposed that the reduction in remanence and coercivity is caused by dipolar interaction and the presence of a magnetic vortex state. Besides, with low concentrations of the soft phase, the exchange interaction on the soft magnetic moments emitted by the hard phase is strong, resulting in the increase in Hc. Meanwhile, by increasing the concentration of the soft phase, the exchange force on soft grains would be enervated. Consequently, the reverse domains in the soft phase with a low nucleation field can be nucleated, resulting in a decline in Hc.

Moreover, Hc is a crucial factor in soft magnetic materials and it can be influenced by diverse defects, particularly a non-homogeneous structure, grain boundaries, dislocations, locally agglomerated nanoparticles, precipitates, pores, airgaps, impurities, the distribution of magnetite nanoparticles in the matrix, and non-magnetic particle dispersion. Accordingly, to reduce Hc, these elements should be kept low [35,36].

This could be the reason that the Hc increased to 165.12 G for Al-30 Fe_3_O_4_. The minimum Hc of 142.34 G for Al-35 Fe_3_O_4_ is the optimum value. Although the addition of more nanoparticles in the composite may result in higher Ms and lower Hc values, increasing the weight percentage of Fe_3_O_4_ can also lead to mechanical degradation. Defects can happen during milling or due to pores and contaminants, resulting in an increased value of Hc. Magnetic properties are investigated at room temperature and the values can be affected by an increase or change in temperature. In general, electromagnetic absorbing materials can be divided into two types based on the loss mechanism, which are dielectric materials SiC and magnetic materials Fe_3_O_4_, and a higher Ms leads to better electromagnetic absorbing properties.

### 3.7. Electrical Resistivity and Conductivity Measurements

**Figure 16:**
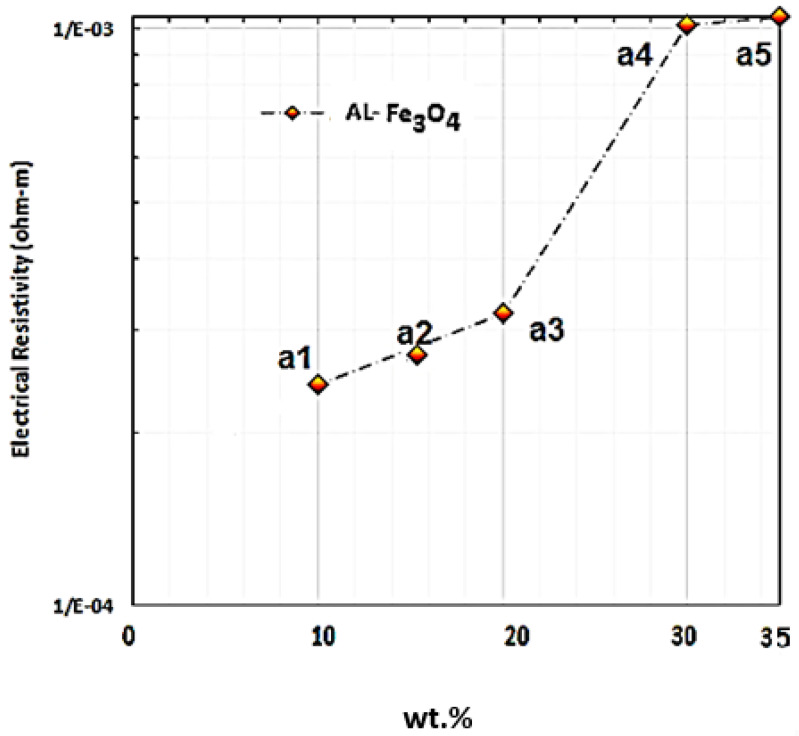
Electric resistivity of Al-Fe_3_O_4_ composites for different weight percentages of Fe_3_O_4_ at room temperature.

The electrical properties of Al-Fe_3_O_4_ were assessed with the four-point probe method. To achieve this objective, DC power was applied with the provided current and voltage of 20 A and 20 V, respectively. Ferreira et al. synthesised an Al-20 Fe_3_O_4_ composite using the microwave method and investigated its electrical resistivity, which was determined to be 0.001028 Ω·m. Figure 16 presents the electrical resistivity of Al-Fe_3_O_4_ composites with different weight percentages of Fe_3_O_4_ into the matrix at room temperature. Based on the experiment, the electrical resistivity of Al-5Fe_3_O_4_ and Al-10 Fe_3_O_4_ are 2 × 10^−4^ and 2.81 × 10^−4^ Ω·m, respectively, and by increasing the weight percentage of Fe_3_O_4_ to 15 and 20 wt.%, the electrical resistivity reached up to 3.21 × 10^−4^ and 4.32 × 10^−4^ Ω·m, respectively. This proved that the electrical resistivity improved slightly with a lower weight percentage of Fe_3_O_4_. By increasing the addition of Fe_3_O_4_ from 20 to 30 wt.%, the electrical resistivity increased significantly to 9.7 × 10^−4^ Ω·m. The highest electrical resistivity is 9.9 × 10^−4^ Ω·m for Al-35Fe_3_O_4_. The decline in the electrical conductivity of the composites can be explained as follows. In producing metal matrix composites, the higher conductivity of reinforcement particles can enhance the electrical conductivity in composites, but for most of the combinations, the particles play a role as an insulator due to the existence of free electrons, which are important for both electrical and thermal conductivity [37]. Due to the free path of an electron, metals can exhibit greater electrical conductivity compared to alloys. The electrical resistivity of Al-Fe_3_O_4_-5% Mg composites increases by adding higher Fe_3_O_4_ content. The electrical resistance of metals illustrates the integrity of the crystal lattice to scatter electron waves. For the conductive properties of Al-Fe_3_O_4_ composites, aluminium is an exceedingly conductive constant phase. The continuous Al matrix determines the electrical conductivity of Al-Fe_3_O_4_ composites and increasing Fe_3_O_4_ particles strongly block the motion of electrons in the matrix, which increases the chance of scattering of electrons. Subsequently, the electrical resistivity of the composite increases. The composite electrical resistivity is related to microstructure-sensitive, nanoparticle arrangement and morphology, and also the number of particles, which significantly influence the resistivity of the material. There is less dispersion of electrons when the distribution of particles in the composite is consistent [38].

As the electrical resistance increases, it can be related to the creation of secondary phases, such as Al_3_Fe, Fe_3_Al, MgAl_2_O_4_, and others. These phases can influence electrical properties adversely. Furthermore, by adding particles, mixed crystals can be formed, leading to lattice deformation and increasing the possibility of electron scattering. The current distorting structure may act in place of insulating materials to inhibit electron tunnelling and transfer from atom to atom, consequently lowering the electrical conductivity of the Al-Fe_3_O_4_ composites. However, it has an insignificant influence on the growth in resistivity. In addition, the agglomeration of Fe_3_O_4_ particles at Al grain boundaries may scatter the charge carrier, and by doing so, the electrical conductivity is reduced. Therefore, the addition of Fe_3_O_4_ decreases the electrical conductivity of aluminium. In electromagnetic shielding or absorption application in aerospace, high electrical conductivity is not essential although it may have a positive effect, where this hybrid composite is classified as a semiconductor material [39].

### 3.8. Measurement of Thermal Properties (Expansion and Conductivity)

The coefficient of thermal expansion “α” explains the change in temperature that leads to the change in object dimensions. The coefficient of thermal expansion of aluminium is high. Hence, by changing the temperature, the dimension of aluminium and its alloy will change, which is approximately two times higher than ferrous metals. As a result, it can cause issues for metallic elements with close tolerances. Conversely, the thermal expansion of ceramic particles is substantially smaller. Thus, it is not unexpected that adding ceramic particles into aluminium will result in a decrease in the thermal expansion of Al-Fe_3_O_4_ composites [40].

**Figure 17:**
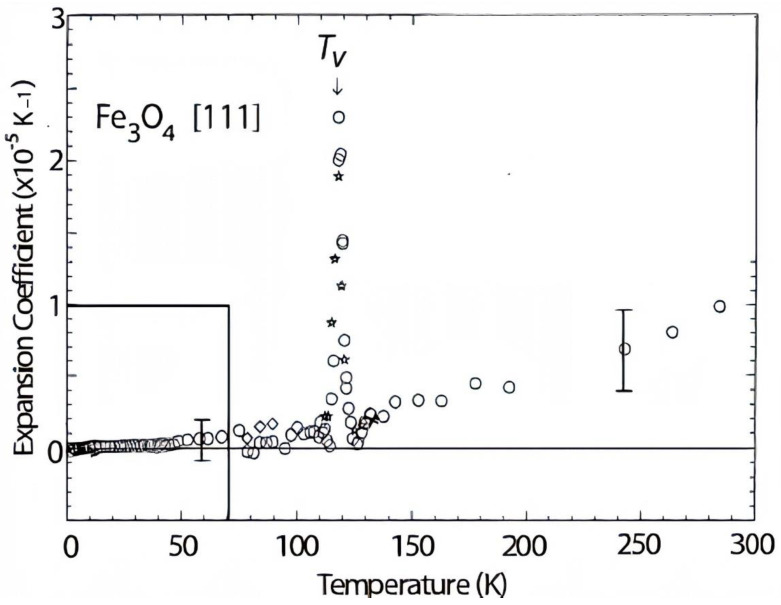
Thermal expansion coefficients of Fe_3_O_4_ at different temperatures, stars refer to the cooling run and circles refer to the heating run (T = 4.2–300 K).

**Figure 18:**
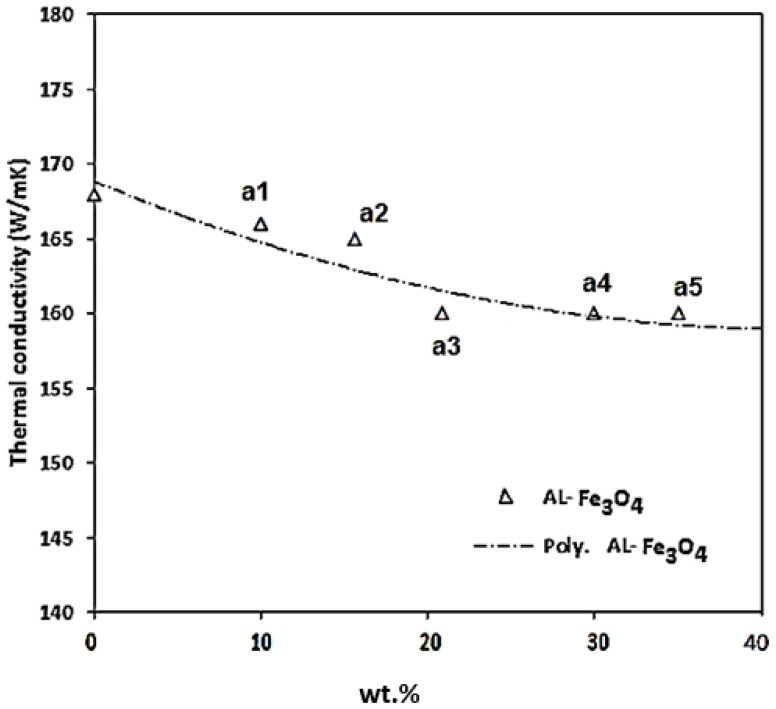
The effect of different weight percentages of Fe_3_O_4_ on the thermal conductivity of Al-Fe_3_O_4_ composites at ambient temperature.

Figure 17 shows the expansion coefficient of Fe_3_O_4_ at different temperatures, where the values increased by raising the temperature. Generally, the thermal diffusivity and thermal conductivity of composites are enhanced by the amplification of temperature. Thermal diffusivity specifies how rapidly a material responds to a variation in temperature and can be evaluated using the laser flash technique. The thermal conductivity of the composites with various weight percentages of Fe_3_O_4_ and pure Al is presented in Figure 18. The test results were obtained at room temperature for Al-Fe_3_O_4_ nanoparticles. The thermal conductivity of all the composites remained almost steady with a low percentage of Fe_3_O_4_. The maximum thermal conductivity is 168 W/mK for Al-5Fe_3_O_4_. The thermal conductivity reduced slightly from 166 to 165 W/mK as the weight percentage of Fe_3_O_4_ increased from 10 to 15 wt.%. As the Fe_3_O_4_ content increased to 20 and 30 wt.%, the thermal conductivity reached 163 and 161 W/mK, respectively. The minimum thermal conductivity is 159 W/mK for Al-35Fe_3_O_4_, which has only a slightly negative influence on these properties, and it is possible that increasing the weight percentage of Fe_3_O_4_ will significantly affect the composites. Fe_3_O_4_ has low thermal conductivity. Therefore, it is necessary to find an optimum weight percentage of Fe_3_O_4_.

A possible explanation of this issue is that lattice vibrations (phonon) and the movement of free electrons conduct the heat in solid materials. By adding Fe_3_O_4_ and Mg particles into aluminium, a higher number of nanoparticles lead to the growth of the interfacial layer and the increase of the interface temperature resistance, resulting in the decline in thermal conductivity. Moreover, the porosity of composites has a complicated effect on thermal conductivity. The pores can be considered as the scattered phase and the increase in porosity will decrease the thermal conductivity [41].

**Figure 19:**
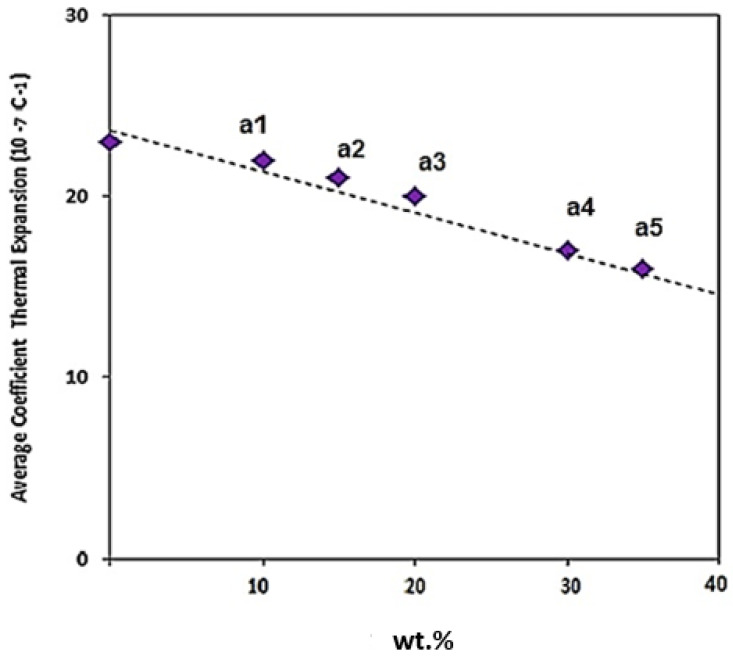
Linear expansion coefficients of the composites with different weight percentages of Fe_3_O_4_.

Figure 19 indicates the linear expansion coefficient of the composites with different weight percentages of Fe_3_O_4_. Based on the results, the thermal expansion coefficient of Al is 2.3 (10^−6^ C^−1^). The Al-5Fe_3_O_4_ composite has a thermal expansion coefficient of 2.2 (10^−6^ C^−1^). As the weight percentage of Fe_3_O_4_ increased from 10 to 15 wt. %, the thermal expansion coefficient decreased from 2.1 to 2.0 (10^−6^ C^−1^), and the thermal expansion coefficient declined from 1.9 to 1.8 (10^−6^ C^−1^) as the weight percentage of Fe_3_O_4_ increased from 20 to 30 wt.%. The thermal expansion coefficient for Al-35Fe_3_O_4_ is 1.7 (10^−6^ C^−1^).

These findings can be explained as follows. First, from Turner’s model, the thermal expansion coefficient of composites mostly correlated with the thermal expansion coefficient of the aluminium matrix and the restriction on the reinforcement through the matrix/reinforcement interface [42]. The main influence on the thermal expansion coefficient is the weight percentage of reinforcement particles. Secondly, interface stress, as a result of the existing mismatch between matrix and particle’s thermal expansion inside the restrictions of the boundary, will cause residual stress in the matrix close to the interfacial area, which can cause the elastic strain in the aluminium matrix. There are other parameters, including the binding force among atoms, grain size, and porosity, that depend on each specification, which may have a negative or positive effect on thermal expansion [43,44].

## 4. Conclusions

Different weight percentages of Fe_3_O_4_ nanoparticles (40 nm) were added into the aluminium matrix to find the optimum weight percentage and to develop magnetic properties using cost-effective powder metallurgy. In accordance with the achieved data and conducted experiments, the five composites have an approximately similar density, from 2.33 g/cm^3^ for Al-10 Fe_3_O_4_ before sintering to 3.29 g/cm^3^ Al-35 Fe_3_O_4_ after sintering. Fe_3_O_4_ nanoparticles were added to aluminium to develop magnetic properties. The results of magnetic properties indicate that the addition of Fe_3_O_4_ from 10 to 15 wt.% in the composite slightly increased the Ms from 2.49 to 6.55 emu/g and reduced the Hc from 231.87 to 188.82 G. By increasing the weight percentage of Fe_3_O_4_ (20–30 wt.%), these values improved significantly to 10.06 and 11.06 emu/g, respectively, and finally reached the maximum Ms of 13.06 emu/g for Al-35Fe_3_O_4_, which is an improvement of 424.5%, whereas the Hc decreased to 142.34 G. The hardness also improved from 43 to 47 HV from the lowest to the highest weight percentage of Fe_3_O_4_ in the composite. The distribution of particles in AMC is homogeneous, which increased the hardness value of aluminium by 9.3%. The thermal conductivity of Al-35Fe_3_O_4_ is 168 W/mk, while for Al-5 Fe_3_O_4_, the thermal conductivity is approximately 159 W/mk. The coefficient of thermal expansion also decreased from 2.2 to 1.7 (10^−6^ C^−1^). Although increasing the weight percentage of Fe_3_O_4_ has a negative effect on thermal conductivity, this value does not change significantly. The electrical resistivity of the Al-35 wt.% Fe_3_O_4_ composite (9.9 × 10^−4^ Ω·m) in the scope of semiconductor materials (10^−10^ Ω·m < δ < 10) can be manufactured by conventional powder metallurgy. Based on the hardness, magnetisation, thermal conductivity, and electrical resistivity of all specimens, the Al-35Fe_3_O_4_ composite can be selected as the optimum composite, particularly for magnetic applications.

## Figures and Tables

**Table 1:** Compositions of aluminium and magnetite composites.

Composition	Al (wt.%)	Fe_3_O_4_ (wt.%)	Mg (wt.%)
Sample a1	85	10	5
Sample a2	80	15	5
Sample a3	75	20	5
Sample a4	65	30	5
Sample a5	60	35	5
